# Manual Dexterity Is a Strong Predictor of Visuo-Motor Temporal Integration in Children

**DOI:** 10.3389/fpsyg.2018.00948

**Published:** 2018-06-12

**Authors:** Satoshi Nobusako, Ayami Sakai, Taeko Tsujimoto, Takashi Shuto, Yuki Nishi, Daiki Asano, Emi Furukawa, Takuro Zama, Michihiro Osumi, Sotaro Shimada, Shu Morioka, Akio Nakai

**Affiliations:** ^1^Neurorehabilitation Research Center, Kio University, Nara, Japan; ^2^Graduate School of Health Science, Kio University, Nara, Japan; ^3^Department of Rehabilitation, Higashiosaka Yamaji Hospital, Osaka, Japan; ^4^Department of Rehabilitation, Nanso-no-Sato, Nursing Care Insurance Facilities, Osaka, Japan; ^5^Department of Home-Visit Rehabilitation, Ishida Clinic, Osaka, Japan; ^6^Department of Rehabilitation, Japan Baptist Hospital, Kyoto, Japan; ^7^Faculty of Education, Kio University, Nara, Japan; ^8^Rhythm-Based Brain Information Processing Unit, RIKEN CBS-TOYOTA Collaboration Center, RIKEN Center for Brain Science, Saitama, Japan; ^9^Department of Electronics and Bioinformatics, School of Science and Technology, Meiji University, Kanagawa, Japan; ^10^Graduate School of Clinical Education & The Center for the Study of Child Development, Institute for Education, Mukogawa Women’s University, Hyogo, Japan

**Keywords:** children, delayed visual feedback detection, developmental change, internal model, manual dexterity, visuo-motor temporal integration

## Abstract

Although visuo-motor temporal integration in children is suggested to be related to motor control and motor learning, its relevance is still unclear. On the other hand, visuo-motor temporal integration ability undergoes developmental changes with age. In the current correlational study, we measured manual dexterity and visuo-motor temporal integration ability in 132 children with typical development (age, 4–15 years) and investigated the relationship between the two functions. The Movement Assessment Battery for Children-2nd edition was used as an indicator of manual dexterity. The delay detection threshold (DDT) and steepness of the probability curve for delay detection, which was measured by the delayed visual feedback detection task for self-generated movement, were used as indices of the visuo-motor temporal integration ability. The results indicated significant correlations between manual dexterity/age and DDT/steepness of the probability curve for delay detection. In addition, hierarchical multiple regression analysis showed that both manual dexterity and age significantly contributed to visuo-motor temporal integration, indicating a better fit than when only age was employed as an independent variable. Importantly, there was no interaction effect between age and manual dexterity. These findings were the first to suggest that manual dexterity is a significant predictor of visuo-motor temporal integration ability in children, regardless of age. The present study validated the important relationship between visuo-motor temporal integration and manual dexterity in children. Considering the limitations of the current study, including the non-homogeneous sample, further studies are still warranted to validate the results.

## Introduction

The visuo-motor integration ability is an important function for motor control and motor learning (Blakemore and Sirigu, 2003; Davidson and Wolpert, 2005). This is largely supported by a neural mechanism well known as the forward or internal model (Wolpert et al., 1995; Kawato, 1999). The internal model provides stability to the motor system by predicting the sensory outcome of movements before actual sensorimotor feedback becomes available, providing a means of rapid online correction (Miall et al., 1993; Wolpert, 1997; Shadmehr et al., 2010; Hyde and Wilson, 2011a,b). When a mismatch occurs between the motor predictions and actual sensory feedback, error signals are thought to be generated in order to correct/modulate the initial movement plan (Miall et al., 1993; Wolpert, 1997; Wolpert et al., 1998a,b; Desmurget et al., 1999; Desmurget and Grafton, 2000; Todorov and Jordan, 2002; Tseng et al., 2007; Shadmehr and Krakauer, 2008). Therefore, integrating the visual feedback with the motor program, that is, visuo-motor integration, is the main function of the internal model.

The visuo-motor integration ability has been reported to be related to the upper limb-hand motor function including manual dexterity. Using the double step reaching task or the motor imagery task, several previous studies demonstrated that the visuo-motor integration in the internal model develops (i.e., improves) during childhood as age increases (Hyde and Wilson, 2013; Wilson and Hyde, 2013; Fuelscher et al., 2015a; Ruddock et al., 2015, 2016). Further, dysfunction of the visuo-motor integration has been reported to result in poor motor functions in children, ensuing conditions such as the developmental coordination disorder (DCD) (Hyde and Wilson, 2011a,b, 2013; Fuelscher et al., 2015b; Ruddock et al., 2015, 2016). Furthermore, the relation between cognitive and motor performance has been previously investigated in 378 children aged 5–6 years (Wassenberg et al., 2005). As a result, specific positive relations were found between manual dexterity and visual motor integration abilities measured by the Beery Developmental Test (Armstrong and Knopf, 1982; Beery, 1997; Wassenberg et al., 2005). There was a significant positive correlation between motor performance and visuo-motor integration, even after controlling for attention, sex, and the presence or absence of signs of psychopathology (Wassenberg et al., 2005). These findings suggested that visuo-motor integration ability develops with age (Hyde and Wilson, 2013; Wilson and Hyde, 2013; Fuelscher et al., 2015a; Ruddock et al., 2015, 2016); however, visuo-motor integration ability decreases if manual dexterity is poor, even at the same age (Fuelscher et al., 2015b; Hyde and Wilson, 2011a,b, 2013; Ruddock et al., 2015, 2016). Further, in children, there is a robust relationship between motor performance and visuo-motor integration (Wassenberg et al., 2005). Therefore, these previous findings indicated an important relationship between visuo-motor integration ability, and age and upper limb-hand motor function (such as manual dexterity), during childhood (Wassenberg et al., 2005; Hyde and Wilson, 2011a,b, 2013; Fuelscher et al., 2015a,b; Ruddock et al., 2015, 2016).

Visuo-motor integration includes a visuospatial aspect and temporal aspect (Makin et al., 2008; Tsakiris, 2010; Kalckert and Ehrsson, 2012, 2014b, 2017). The double step-reaching task (Hyde and Wilson, 2013; Wilson and Hyde, 2013; Fuelscher et al., 2015a,b; Ruddock et al., 2015, 2016) and the Beery Developmental Test (Wassenberg et al., 2005) used in previous studies reflect both the visuospatial and temporal aspects of the visuo-motor integration. The current study focused on the developmental changes of the temporal aspect of the visuo-motor integration. The temporal aspect of visuo-motor integration involves motor coordination such as timing, rhythm, and tempo, which is more essential to the generation of sense of agency than the spatial visuo-motor aspects (Kalckert and Ehrsson, 2012, 2014a). However, whether manual dexterity in children has a significant influence on visuo-motor temporal integration is still unknown.

Jaime et al. (2014) investigated the developmental changes of the visuo-motor temporal integration ability in children aged 5–8 years and in adults aged 18–22 years using the visual feedback delay detection task. In this previous study, children were instructed to observe a monitor displaying their movements of a joystick at varying delay durations and to make judgments on whether their movements appeared to be delayed or live. As a result, a decrease in the delay detection threshold (DDT) was observed with increasing age of the participants. Furthermore, the authors analyzed the speed of moving the joystick, which indicated that there was no relation between movement speed and DDT. These previous results indicated a specific improvement of the visuo-motor temporal integration ability with age. However, this previous study did not consider the relationship between manual dexterity and visuo-motor temporal integration in children.

Based on previous studies reporting on the significant relationship between visuo-motor integration and manual dexterity in children (Wassenberg et al., 2005; Hyde and Wilson, 2011a,b, 2013; Wilson and Hyde, 2013; Fuelscher et al., 2015a,b; Ruddock et al., 2015, 2016), the visuo-motor temporal integration in children is expected to be related not only to age but also to motor control functions such as manual dexterity. Therefore, the current study investigated the relationship between the ability to integrate visuo-motor information temporally and age and manual dexterity in children. We hypothesized that the visuo-motor temporal integration ability in children would increase with age, similarly to previous findings (Jaime et al., 2014), together with motor control function improvement.

To verify this hypothesis, we used a delay in the visual feedback task similar to the one used in a previous study (Jaime et al., 2014). However, this task involved self-generated movement and visual feedback that were both perceived at two different locations, that is, the movement occurred at one location and visual feedback of the same movement was displayed on a screen placed at another location. Thus, there was a possibility that not only the pure temporal aspect of the visuo-motor integration but also the visuospatial aspect was included in this task; consequently, the DDT was likely to be affected by both temporal and spatial errors. To prevent such spatial errors, the task in the current study employed the experimental settings used by Shimada et al. (2010), who used a double-sided mirror and delay-inserting device to present the delayed hand image of the participant’s own hand position. During the task, the participant reported if the visual feedback was synchronized (no delay) or not (delayed) with the self-generated hand movement. In addition, Jaime et al. (2014) only used four delayed visual feedback time-points (i.e., 0, 100, 200, and 300 ms). In the video delay system used in the current study (Shimada et al., 2010), it was possible to generate a minimum delay of 33.3 ms. Indeed, setting finer delay intervals leads to more detailed evaluation of the visuo-motor temporal integration ability (Shimada et al., 2010). Therefore, the current study was based on 18 delay time-points from 33 to 600 ms at intervals of 33.3 ms.

The DDT (time delay in ms) indicates the extent to which the brain allows a temporal discrepancy between different sensory modalities, including motor signals (efference copy). The steepness of the delay detection probability curve, which will be referred to as steepness from this point onward, indicates the mechanism by which the brain integrates multisensory signals; thus, the steepness would be steeper if the judgment is more strict and precise (Shimada et al., 2010). Therefore, decreasing DDT and increasing steepness represent a highly sensitive visuo-motor temporal integration. Thus, both DDT (when the rate of delay detection is 50%) and the steepness served as indices of visuo-motor temporal integration in the present study. Considering the results described by Shimada et al. (2010) and Jaime et al. (2014), we hypothesized that DDT would decrease and steepness would increase with age and motor control improvement.

The Movement Assessment Battery for Children-2nd edition (M-ABC-2) (Henderson et al., 2007), which is an international assessment battery of manual dexterity in children, was considered optimal for the current study since it is a standardized age-adjusted test and was used to measure the hand motor control function. Therefore, the hand motor control function measured in the current study was expressed as “manual dexterity” according to the M-ABC-2.

Jaime et al. (2014) demonstrated developmental changes of the visuo-motor temporal integration ability between 5 and 8-year-old children and 18 and 22-year-old adults. At the level of movement skills, previous reports indicated a gradual increase in reaching proficiency from mid childhood (∼5 years) into early adulthood (Bard et al., 1990; Chicoine et al., 1992). Therefore, we recruited a broader age-range between 4 and 15 years in the present study to investigate the developmental course of the relationship between the visuo-motor temporal integration ability and manual dexterity.

## Materials and Methods

### Participants

A total of 139 children participated in this study. Of these, seven children were excluded from the analysis since they could not complete the experiment. The remaining 132 children [mean age ± standard deviation (SD), 8.9 ± 2.5 years; range, 4–15 years; 86 male participants; 114 right-handed] completed the manual dexterity test and the delayed visual feedback detection task. Children were recruited to participate in this study from public preschools (nursery school and kindergarten), public primary schools, and public secondary schools in Osaka, Japan. Only children with typical development who were enrolled in regular classes were recruited. The exclusion criteria consisted of the following: (1) a general medical condition (e.g., cerebral palsy, hemiplegia, and muscular dystrophy), (2) diagnosis of a developmental disorder (e.g., DCD, autism spectrum disorder, attention deficit hyperactivity disorder, learning disorder, and pervasive developmental disorder), or (3) diagnosis of intellectual disability. Eligibility was confirmed by interviewing parents and the results of the regular checkup, which were provided by the school doctor at each school. The study was approved by the local ethics committee and parents of the participating children provided informed consent. **Table 1** summarizes the participants’ age, sex, and preferred hand distributions.

**Table 1:** Distributions of age, sex, and preferred hand of the participants.

Age (years)	Number	Sex		Preferred hand	
			
		Male (*n*)	Female (*n*)	Right (*n*)	Left (*n*)
4	2	0	2	2	0
5	11	10	1	10	1
6	8	3	5	4	4
7	24	16	8	21	3
8	20	16	4	16	4
9	22	11	11	19	3
10	9	6	3	9	0
11	11	9	2	8	3
12	11	6	5	11	0
13	8	4	4	8	0
14	5	4	1	5	0
15	1	1	0	1	0
Total	132	86	46	114	18


### Procedures

The ages of each of the children were recorded. Children were subjected to the manual dexterity test and the delayed visual feedback detection task in prescribed rooms of each preschool or school. The area of prescribed rooms at each school was approximately 70 m^2^. The time required to complete each test was less than 30 min, and both tests were completed within 1 h for each participant. The two tests were performed in a random order for each child. Randomization was performed by replacement block method using the RAND function of Excel (Microsoft Excel, 2016). The two tests were not blinded. During the tests, only the child and the experimenters were present in the prescribed room. However, if the parents or school teachers wished to attend the sessions, they were then permitted in the room.

#### Manual Dexterity

The manual dexterity test of the M-ABC-2 (Henderson et al., 2007) is a standardized age-adjusted test used to identify motor deficits in children using different tasks for different age bands. The M-ABC-2 has good test-retest reliability (minimum value at any age is 0.75), good inter-rater value (0.70), and good concurrent validity (Henderson et al., 2007). This test has three age bands encompassing the following age ranges: 3–6 years (age band 1), 7–10 years (age band 2), and 11–16 years (age band 3). In the current study, each child received three sub-tests that were appropriate for his/her age band. Age band 1 (3–6 years) was evaluated by the posting coins test, threading beads test, and drawing trail I test. Age band 2 (7–10 years) was evaluated by the placing pegs test, threading lace test, and drawing trail II test. Age band 3 (11–16 years) was evaluated by the turning pegs test, triangle with nuts and bolts test, and drawing trail III test. Based on the examiner’s manual of M-ABC-2, the standard scores were calculated from the raw scores. The standard score, which reflects the degree of manual dexterity for each age year, was used as an index of manual dexterity in the current study. Thus, increases in the standard score represented improvement in manual dexterity within each age year. All the assessments were administrated by a specifically trained and certified physical therapist. (The physical therapist completed the M-ABC-2 Japanese version development project program in 2015, which was hosted by Prof. Akio Nakai, the Japanese version copyright holder of M-ABC-2. In the present study, the M-ABC-2 was used as collaborative research with Prof. Akio Nakai.)

#### Delayed Visual Feedback Detection Task

##### Materials and equipments

This task required a video camera, converter, video delay device, monitor, and two-sided mirror. The video camera (FDR-AXP35, Sony, Tokyo, Japan) was used to record the hand motion of children using the following parameters: 1,920 × 1,080 pixels, a bit rate of 50 Mbps, and frequency of 25 MHz. The High Definition Multimedia Interface (HDMI) cable was connected to the video camera and converter (HA5 Mini-Converter, AJA Video Systems, California, United States); the Serial Digital Interface (SDI) cables connected the converter, video delay device, and monitor. The converter was used to convert the HDMI signal captured by the video camera into the SDI signal. The video delay device (EDS-3306, FOR-A YEM ELETEX, Tokyo, Japan) inserted a time delay into the SDI signal from the converter, which was sent to the monitor as an SDI signal. This delay device inserted a delay at intervals of 33.3 ms for moving pictures. The monitor (LMD-A240, Sony, Tokyo, Japan) that displayed the delayed video had a screen size of 24.1″ and a resolution of 1,920 × 1,200 pixels. The double-sided mirror that reflected the moving image from the monitor had a size of 45 cm × 45 cm.

##### Experimental setup

**FIGURE 1:**
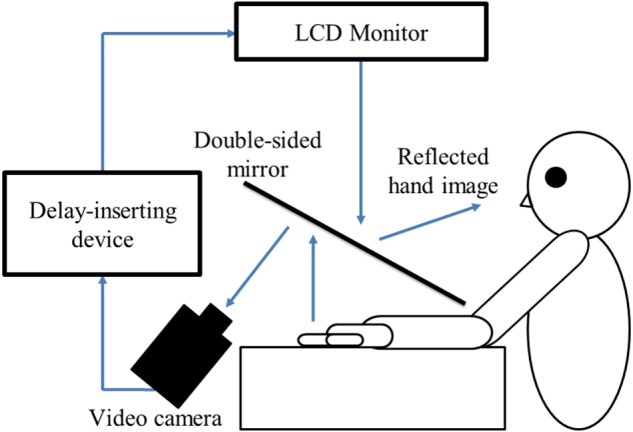
Experimental setup and experimental task. The children were instructed to place their preferred hand under the two-way mirror. Subsequently, the children opened and closed their preferred hand by their own volition after the experimenter had orally informed them of the start of a trial. Their preferred hand movement was filmed by a video camera. Visual feedback delay was achieved using a hardware device. The children observed the reflected image of their delayed hand movement displayed on an LCD monitor. Subsequently, they were instructed to reply orally “delayed” or “not delayed” by the forced-choice method immediately following each trial.

In this study, a similar experimental setup as Shimada et al. (2010) was used (**Figure 1**). The child’s preferred hand was placed under a two-way mirror, so the child was unable to directly see his/her hands. The image of the hand, which was reflected in the two-way mirror, was filmed with a video camera. The movie of the photographed hand was further reflected from an installed monitor onto the two-way mirror via a video delay device. Thus, the child observed the delayed image of their own hand reflected in the mirror at the position where their own hand would be. The dimensions of the experimental setup were 45 cm × 60 cm × 70 cm (length × width × height), and the shape was a cube. In addition, the setup included a blackout curtain so that the child would not be able to see the experimenter. However, the side where the child is sitting was unobstructed and opened. Therefore, the children were not trapped in a narrow space. The intrinsic delay of the visual feedback in this experimental setting was 33.7 ms as measured by a time lag check device (EDD-5200, FOR-A YEM ELETEX, Tokyo, Japan).

##### Experimental task

The delayed visual feedback detection task was performed using the preferred hand of each child. Children were instructed to observe the reflection in the mirror with the following instruction: “Please observe your own hand reflected in the mirror.” Subsequently, children opened and closed their hand once continuously and smoothly based on the child’s own volition after the experimenter had orally informed them of the start of a trial. Given that no relationship between the reaction time/movement speed and visuo-motor temporal integration ability has been demonstrated previously (Jaime et al., 2014), we did not record the reaction time and movement speed in the present study.

With regard to the self-generated movement, 18 delay conditions were set by using a video delay-inserting device: 33, 67, 100, 133, 167, 200, 233, 267, 300, 333, 367, 400, 433, 467, 500, 533, 567, and 600 ms. In the study by Jaime et al. (2014), the four delay conditions (0, 100, 200, and 300 ms) indicated that the DDT at ages ranging from 5 to 8 years was approximately 100–300 ms. Since the children in the present study were 4–15 years old, the delay conditions were broadly set at 33–600 ms. In addition, in order to extract the visuo-motor temporal integration ability in more details, we set the 18 delay conditions at 33.3-ms intervals. All the 18 delay conditions were treated as one set, performed four times, and their presentation order was randomized. A random order table was created using the RAND and rearrangement functions of Excel (Microsoft Excel, 2016) for each participating child in advance. The task was performed according to the random order table that was created. Thus, each child completed a total of 72 randomized trials with 18 delay conditions per four sets, which was consistent with previous studies (Shimada et al., 2005, 2010, 2014).

During the delayed visual feedback detection task, the children were only looking at the hand on the mirror, but not the real hand. Thus, children felt their own hand moving while watching the display of the delayed mirror reflection of that same movement. Each child had to determine if the visual feedback was synchronous or asynchronous relatively to the preferred hand movements performed based on their own intention. Immediately following the trial, the child had to state orally by the forced-choice method, that is, “delayed” or “not delayed.” A 10-s rest time was set between each trial.

The delayed visual feedback detection task was conducted after sufficient explanation and practice to ensure that the children adequately understood the task. Specifically, first of all, the children received an explanation that this task has no correct answers and no false answers. In other words, the experimenter thoroughly explained to each child that they could answer with free subjective judgment. In addition, the children practiced alternately repeating, with a minimum delay of 33 ms and maximum delay of 600 ms. At that time, whichever the child’s report was “delayed” or “not delayed,” the experimenter replied “OK.” Furthermore, before the task, each child confirmed that they could distinguish between a minimum delay of 33 ms and a maximum delay of 600 ms. That is, before the task, all children reported “not delayed” for the minimum delay of 33 ms and reported “delayed” for the maximum delay of 600 ms. In doing so, the experimenter responded “OK” to all the children’s reports, without giving feedback of whether the participant’s answers were correct or incorrect. Further, during the task, the experimenter also replied “OK” to all the children’s answers.

##### Experimental task data analysis

In order to examine the differences in the determination curve shapes of each child, the logistic curve was fitted to the child’s responses on the visual feedback delay detection task (Afraz et al., 2006; Shimada et al., 2010) using the following formula: *P(t) = 1/1+exp(-a(t-DDT))*, where *t* is the visual feedback delay length, *P(t)* is the probability of delay detection, *a* indicates the steepness of the fitted curve, and *DDT* indicates the observer’s DDT that represents the delay length at which probability of delay detection is 50%. In our experiment, *t* served as an independent variable, while *P(t)* was the observed value. The curve was fitted using a non-linear least squares method (a trust-region algorithm) with the Curve Fitting toolbox in Matlab R2014b (MathWorks Inc., Natick, MA, United States) to estimate *a* and *DDT*.

### Statistical Analysis

All statistical analyses were performed using SPSS ver. 24 (SPSS, Chicago, IL, United States). The data collected, that is, age, manual dexterity (standard score), and results of the experimental tasks, were analyzed using correlation and hierarchical multiple regression analyses.

#### Correlation Analysis

Using the Shapiro–Wilk test, a normal distribution was observed in DDT, but not in age, manual dexterity, and steepness. Therefore, the statistical analysis was performed using the Spearman’s correlation coefficient by rank test for the purposes of testing the correlation between DDT, steepness, age, and manual dexterity. The significance level was set at *p* < 0.05.

#### Hierarchical Multiple Regression Analysis

A hierarchical multiple regression analysis (forced entry method), in which DDT and steepness were the dependent variables and age, manual dexterity, and age × manual dexterity interaction term were the independent variables, was also performed. The interaction term of age and manual dexterity was calculated by multiplying the value obtained by centering each variable of age and manual dexterity in consideration of multicollinearity. In model 1, age was the independent variable (regression equation: *DDT (steepness) = a + (b1 × age)*]. In model 2, manual dexterity was added to model 1 as an independent variable [regression equation: *DDT (steepness) = a + (b1 × age) + (b2 × manual dexterity)*]. In model 3, an interaction term was added to model 2 as an independent variable with the following regression equation: *DDT (steepness) = (a + [b2 × manual dexterity]) + (b1 + [b3 × manual dexterity]) × age*. In the regression equation, *a* represents the intercept, and *b* represents the non-standardized partial regression coefficient. The level of significance was set at *p* < 0.05.

## Results

**Table 2** summarizes the data on age, manual dexterity (standard score), DDT, and steepness in the participating children.

**Table 2:** Distributions of age, manual dexterity, and visuo-motor temporal integration ability of the participants.

	Age (years)	Manual dexterity (standard score)	Visuo-motor temporal integration ability	
			
			DDT (ms)	Steepness
Mean	8.9	9.9	263.6	0.072
Standard deviation	2.5	3.8	104.2	0.113
Range	4–15	2–19	66–546.3	0.003–0.556
Skewness	0.34	–0.30	0.39	2.77
Kurtosis	–0.62	–0.67	–0.19	7.61


*DDT, delay detection threshold.*

### Correlation Analysis Results

**FIGURE 2:**
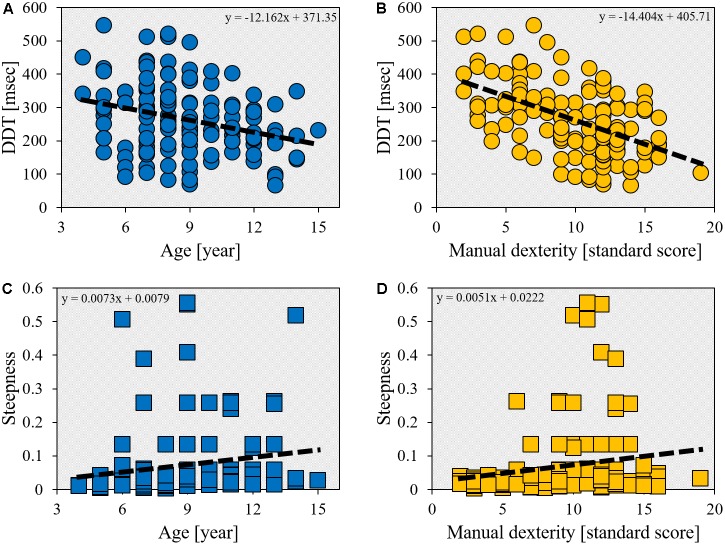
Scatter plot showing the relationship between age/manual dexterity and delay detection threshold (DDT)/steepness. The horizontal axis shows age (year) or manual dexterity (standard score). **(A)** The correlation between DDT and age (*r* = -0.283, *p* = 0.001, *n* = 132). **(B)** The correlation between DDT and manual dexterity (*r* = -0.497, *p* < 0.001, *n* = 132). **(C)** The correlation between steepness and age (*r* = 0.348, *p* < 0.001, *n* = 132). **(D)** The correlation between steepness and manual dexterity (*r* = 0.234, *p* < 0.007, *n* = 132).

The DDT was significantly inversely correlated with age (*r* = -0.283, *p* = 0.001; **Figure 2A**). In addition, there was a significant inverse correlation between DDT and manual dexterity (*r* = -0.497, *p* < 0.001; **Figure 2B**). The steepness was also significantly correlated with age (*r* = 0.348, *p* < 0.001; **Figure 2C**) and manual dexterity (*r* = 0.234, *p* = 0.007; **Figure 2D**). Furthermore, age and manual dexterity (*r* = -0.184, *p* = 0.034), in addition to DDT and steepness (*r* = -0.439, *p* < 0.001), were also significantly inversely correlated.

### Hierarchical Multiple Regression Analysis Results

The results of the hierarchical multiple regression analysis are summarized in **Table 3**. For DDT, the coefficient of determination adjusted for the degrees of freedom (Adjusted R^2^) was the highest, the multiple coefficient of determination (R^2^) was significantly changed, and Akaike information criterion (AIC) and Bayesian information criterion (BIC) were the lowest (meaning the best fit) when both age and manual dexterity (model 2) were employed as independent variables, compared to model 1 (age only) and model 3 (interaction). In addition, there was no interaction effect between age and manual dexterity. Similar results were obtained for the steepness, indicating that model 2 was the best regression model to predict visuo-motor temporal integration abilities.

**Table 3:** Hierarchical multiple regression analysis results.

Dependent variable	Model	Independent variable	Partial regression coefficient (B)	Standardized regression coefficient (β)	*p*-value	VIF	AIC	BIC
DDT	1	(constant)	263.642		<0.001		1218.398	1224.164
		Age (year)	-12.162	-0.297	0.001	1.000		
		*R* = 0.297, *R*^2^ = 0.088, Adjusted *R*^2^ = 0.081, *p* = 0.001						
	2	(constant)	263.642		<0.001		1161.080	1169.729
		Age (year)	-16.044	-0.391	<0.001	1.027		
		Manual dexterity^a^	-16.171	-0.582	<0.001	1.027		
		*R* = 0.647, *R*^2^ = 0.418, Adjusted *R*^2^ = 0.409, *p* < 0.001; Δ*R*^2^ = 0.330, Δ*F* = 73.186, *p* < 0.001						
	3	(constant)	264.883		<0.001		1162.138	1173.669
		Age (year)	-16.082	-0.392	<0.001	1.027		
		Manual dexterity^a^	-15.927	-0.574	<0.001	1.046		
		Interaction effect^b^	0.800	0.065	0.340	1.020		
		*R* = 0.650, *R^2^* = 0.422, Adjusted *R^2^* = 0.409, *p* < 0.001; Δ*R*^2^ = 0.004, Δ*F* = 0.004, *p* = 0.340						
Steepness	1	(constant)	0.072		<0.001		-576.169	-570.404
		Age (year)	0.007	0.164	0.060	1.000		
		*R* = 0.164, *R^2^* = 0.027, Adjusted *R^2^* = 0.019, *p* = 0.060						
	2	(constant)	0.072		<0.001		-579.629	-570.981
		Age (year)	0.009	0.197	0.024	1.027		
		Manual dexterity^a^	0.006	0.201	0.021	1.027		
		*R* = 0.258, *R^2^* = 0.066, Adjusted *R^2^* = 0.052, *p* = 0.012; Δ*R*^2^ = 0.039, Δ*F* = 5.448, *p* = 0.021						
	3	(constant)	0.074		<0.001		-578.599	-567.068
		Age (year)	0.009	0.196	0.025	1.027		
		Manual dexterity^a^	0.006	0.213	0.016	1.046		
		Interaction effect^b^	0.001	0.083	0.333	1.020		
		*R* = 0.271, *R^2^* = 0.073, Adjusted *R^2^* = 0.051, *p* = 0.021; Δ*R*^2^ = 0.007, Δ*F* = 0.944, *p* = 0.333						


*^*a*^Manual dexterity is represented by the standard score; ^*b*^Interaction effect is represented by Age × Manual dexterity. AIC, Akaike information criterion; BIC, Bayesian information criterion; VIF, variance inflation factor.*

The detailed results of the hierarchical multiple regression analysis (model 2) was as follows. With DDT as a dependent variable, both age (β = -0.391, *p* < 0.001) and manual dexterity (β = -0.582, *p* < 0.001) were significant independent variables. The relationship between DDT and age/manual dexterity could be modeled with the following equation: DDT = 263.642 + (–16.044 × age) + (-16.171 × manual dexterity), resulting in the following results: *R* = 0.647, *R*^2^ = 0.418, Adjusted *R^2^* = 0.409, *p* < 0.001. Importantly, age and manual dexterity did not have multicollinearity.

With steepness as a dependent variable, both age (β = 0.197, *p* = 0.024) and manual dexterity (β = 0.201, *p* = 0.021) were significant independent variables. The relationship between steepness and age/manual dexterity can be modeled with the following multiple regression equation: steepness = 0.072 + (0.009 × age) + (0.006 × manual dexterity), resulting in the following results: *R* = 0.258, *R*^2^ = 0.066, Adjusted *R^2^* = 0.052, *p* = 0.012. Age and manual dexterity did not have multicollinearity.

## Discussion

In this study, the standard score measured by M-ABC-2 was used as an index of manual dexterity, while DDT and steepness measured by delayed visual feedback task were used as indices of visuo-motor temporal integration to investigate the developmental change of the temporal aspect of visuo-motor integration in 4–15-year-old children. The correlation analysis results showed that both age and manual dexterity were significantly correlated with the visuo-motor temporal integration ability. Thus, not only the visuo-motor temporal integration ability improves as age increases, but also the visuo-motor temporal integration ability improves as the manual dexterity improves. Both DDT and steepness showed a negative correlation in the current study. Thus, the shorter the time window of the visuo-motor temporal integration, the more strict and precise was the delayed detection, and vice versa. In addition, the hierarchical multiple regression analysis revealed that both age and manual dexterity were significant independent variables for DDT and steepness as the dependent variables. However, there was no interaction effect between age and manual dexterity. Therefore, age and manual dexterity were each independently, but not together, a significant predictor of visuo-motor temporal integration.

### Relationship Between Visuo-Motor Temporal Integration and Age

Based on our results, both DDT and steepness were significantly correlated with age. Furthermore, the hierarchical multiple regression analysis revealed that age significantly predicted the visuo-motor temporal integration ability, which showed improvement with age. This finding corroborated with the outcomes of previous study (Jaime et al., 2014) that demonstrated developmental changes of the visuo-motor temporal integration ability with advancing age. However, unlike the previous study (Jaime et al., 2014) that involved not only the pure temporal aspect but also the visuospatial aspect, the present study used the task of Shimada et al. (2010), which eliminated the visuospatial aspect and extracted pure temporal aspect. This was because the present study focused on the relationship between the manual dexterity and the temporal aspects of visuo-motor integration in children. Furthermore, the present study addressed the possibility issue of including the visuospatial aspect in the previous experimental method (Jaime et al., 2014). Therefore, we believe that the present data reflects more accurately the visuo-motor temporal integration ability in children. We additionally performed correlation analysis between age and visuo-motor asynchrony detection ability, thus provided direct support and additional evidence for this correlation. It has been demonstrated that visuo-motor integration improves with increasing age (Hyde and Wilson, 2013; Wilson and Hyde, 2013; Fuelscher et al., 2015a; Ruddock et al., 2015, 2016). For example, Wilson and Hyde (2013) demonstrated that online corrections in the double-step reaching task were faster in older and mid-aged children (8–12 years) compared to younger children (6–7 years). Consistent with previous findings, our current results provided additional evidence that the temporal aspects within the visuo-motor integration also improved with age.

### Relationship Between Visuo-Motor Temporal Integration and Manual Dexterity

There was a significant correlation between manual dexterity and DDT/steepness, whereby visuo-motor temporal integration ability improved as manual dexterity improved. The hierarchical multiple regression analysis indicated that the predictability of the statistical model was better when both age and manual dexterity were set as independent variables than when only age was set as an independent variable. Furthermore, the hierarchical multiple regression analysis also revealed no interaction between age and manual dexterity. Thus, age and manual dexterity were each independently significant independent predictors of visuo-motor temporal integration. The current study is novel as it indicates that differences in hand skills of children are related to their ability to integrate movement and visual feedback in a temporal sequence, regardless of age. The M-ABC-2 provided the age-adjusted standard scores for each year between 4 and 16 years, thus canceling the effects of age differences; the increased standard score with increasing age indicated a positive correlation. However, based on our results, the M-ABC-2 standard score (manual dexterity) did not increase with age. In addition, the hierarchical multiple regression analysis indicated that age and manual dexterity did not have multicollinearity problems. Therefore, the possibility that the visuo-motor temporal integration has improved simply by improving manual dexterity with increasing age can be excluded. Furthermore, the missing interaction effect between age and manual dexterity suggested that manual dexterity exerted a main effect (direct effect) on visuo-motor temporal integration, independently of age. Thus, the current results suggested that age and manual dexterity contributed to visuo-motor temporal integration via different mechanisms.

The integration of self-generated movement and visual feedback is crucial in motor control. Indeed, it has already been demonstrated that a dysfunctional visuo-motor integration in the developmental process leads to motor coordination disorders (Hyde and Wilson, 2011a,b, 2013; Fuelscher et al., 2015b; Ruddock et al., 2015, 2016). Fuelscher et al. (2015a) demonstrated that the correction time for movement in the double-step reaching task becomes faster from younger children to older children to adolescents. This previous study (Fuelscher et al., 2015a) revealed that visuo-motor integration ability improves with age. However, the movement’s correction time was delayed in children with poor motor skills such as DCD even when compared with children from the same age group (8–12 years) (Fuelscher et al., 2015b), indicating that differences in motor function affect visuo-motor integration, even at the same age. Our current results corroborated these previous findings and revealed that manual dexterity is a significant predictor of visuo-motor temporal integration ability, regardless of age.

On the other hand, our current results suggested that lower visuo-motor temporal integration ability may lead to poor manual dexterity. Comparing motor predictions with actual sensory feedback, generating error signals and correcting the motor commands online is an important role of the internal model. Importantly, error signals also act as training signals to refine the accuracy of predictive models. This iterative process is thought to be fundamental for motor learning (Davidson and Wolpert, 2005). Thus, a major mismatch in motor predictions/actual proprioceptive feedback and actual visual feedback during the initiation of self-generated movement can cause unsuccessful movement. In other words, the reduced ability of visuo-motor temporal integration impedes the generation of error signals, which may cause movement failure. Using writing, drawing, star, or maze tracing tasks (Smith et al., 1960), steering (Smith and Kaplan, 1970), manual tracking (Miall et al., 1985), pegboard task (Fujisaki, 2012), reaching task (Botzer and Karniel, 2013), and sequential motor task (Kulpa and Pfordresher, 2013), previous studies demonstrated that artificially delaying the visual feedback from a self-generated hand movement decreased the hand motor performance, thus hampering the adaptive motor learning. Moreover, the delay in visual feedback slowed the rate and extent of prism adaptation (Kitazawa et al., 1995; Tanaka et al., 2011) and decreased muscle activity (Imaizumi et al., 2014). In a recent report, the delay of visual feedback from self-generated hand movement resulted in dysesthesia (i.e., decreased limb ownership and increased heaviness) in addition to the reduction of muscle activity (Osumi et al., 2017). These studies suggested that the diminished ability to integrate the hand movement and visual feedback in a temporal sequence may cause a decrease in manual dexterity. Our present results corroborated these previous findings.

Several previous studies revealed that the motor imagery ability is an important predictor of the development of visuo-motor integration (Wilson and Hyde, 2013; Fuelscher et al., 2015a,b). Taken together with our current results, these findings suggested that age, manual dexterity, and motor imagery ability contributed to the development of visuo-motor temporal integration, and that these elements are associated bidirectionally.

## Conclusion

The current study investigated the link between manual dexterity and visuo-motor temporal integration in 132 children aged between 4 and 15 years. The current results revealed that manual dexterity is a significant predictor of visuo-motor temporal integration regardless of age. However, the mechanisms underlying this link are yet to be elucidated.

### Limitations and Future Directions

Several limitations in the present study are worth noting. First, the children included in the study did not have a homogeneous distribution in age and manual dexterity. Therefore, comparisons between the age groups without a difference in manual dexterity, and comparisons between the manual-dexterity groups without a difference in age were difficult. As a result, this study was limited to correlational analyses, and the current results remained a limited contribution to the developmental change of visuo-motor temporal integration.

Second, although the delayed visual feedback detection task was performed after sufficient explanation and practice to ensure that the children adequately understood the task, the current study did not measure objectively the children’s attention (e.g., using an eye tracker), which could affect the visuo-motor temporal integration. In the future, it is necessary to provide evidence that children are observing the reflection of their own hand during the task.

Third, in the previous study (Jaime et al., 2014), since there was no relationship between the reaction time/movement speed and visuo-motor temporal integration ability in the experimental task, the current study did not measure the reaction time/movement speed in the experimental task. However, these measurements may have resulted in new findings on the relationship between manual dexterity and reaction time/movement speed in the experimental task. Therefore, in future studies, it is crucial to measure the reaction time/movement speed in the experimental task.

Fourth, the children’s intelligence quotient (IQ), which could have affected the current results, was not measured in this study. Nonetheless, the recruited children attended regular classes at public preschools, primary schools, or secondary schools, and exhibited a typical development, without general medical conditions, developmental disorders, or intellectual disabilities. Thus, it was assumed that the IQ did not affect the current results; however, future studies need to implement the IQ in their measures to yield more definitive conclusions.

Fifth, although our results indicated a significant relationship between visuo-motor temporal integration and manual dexterity, self-generated movement consisted of motor predictions and proprioceptive feedback. Thus, it remains unclear whether or not the visuo-motor temporal integration measured in the present study indicated the ability to integrate motor predictions and visual feedback or the ability to integrate proprioceptive and visual feedback. Further research that clearly distinguishes between passive (delayed visual feedback from proprioception task) and active movement (delayed visual feedback from active movement task) is required.

Sixth, the substantial relationship between visuo-motor temporal integration and manual dexterity in children suggested that the diminished ability to integrate movement and visual feedback in a temporal sequence leads to the loss of manual dexterity. DCD is a typical developmental disorder characterized by clumsy manual dexterity. The current study did not classify the included children into DCD and typical development. However, based on the manual dexterity M-ABC-2 test scores, there were 20 children (mean percentile ± SD, 2.3 ± 1.8; percentile range, 0.5–5th percentile; mean age ± SD, 8.7 ± 2.0 years; age range, 5–14 years; 18 male participants; 16 right-handed) who scored in the 5th percentile or less. Thus, these 20 participants had probable DCD. However, none of the participants had received a formal DCD diagnosis. Therefore, although this idea is speculative, autonomic nervous functions (such as fatigue and stress) and childish feelings (childish phobic reactions) such as claustrophobia and nyctophobia, may have influenced the results of the present study (M-ABC-2, Experimental task). Although none of the 132 participants interrupted participation in experiments due to fatigue or fear, the present study included 21 children in the early childhood age group and did not objectively measure fatigue or fear. Therefore, future studies should include objective measurements of the listed autonomic nervous functions and childish feelings, to lead to a better understanding of the relationship between motor function in children, visuo-motor integration, and psychological aspects.

Seventh, the present results suggested that the decrease in visuo-motor temporal integration ability could lead to poor manual dexterity. It has already been demonstrated that DCD is characterized by deficits of the visuo-motor integration in the internal model (Hyde and Wilson, 2011a,b, 2013; Fuelscher et al., 2015b; Ruddock et al., 2015, 2016; Nobusako et al., 2018). The current results and these previous studies suggest that improvement of visuo-motor integration may improve the clumsiness of movement in children. Therefore, future studies on habilitation to improve visuo-motor integration are necessary.

## Ethics Statement

The experimental procedures were approved by the local ethics committee of the Graduate School and Faculty of Health Sciences at Kio University (approval number: H27-33). There were no foreseeable risks to the participants, and no personally identifying information was collected. Participants (child’s parents) provided background information and gave written informed consent. Before starting the study, it was explained to participants that they could withdraw from the study at any time, without receiving any disadvantageous treatment. Withdrawal of consent was possible at any time by oral retraction of parents or children. The procedures complied with the ethical standards of the 1964 Declaration of Helsinki regarding the treatment of human participants in research.

## Author Contributions

SN collected and analyzed the data and wrote the manuscript. AS, TT, TS, and EF assisted in collecting data. SN designed the study. YN, DA, EF, TZ, MO, SS, SM, and AN designed and supervised the study. All authors read and approved the manuscript.

## Conflict of Interest Statement

The authors declare that the research was conducted in the absence of any commercial or financial relationships that could be construed as a potential conflict of interest.
